# Across the adult lifespan the ipsilateral sensorimotor cortex negative BOLD response exhibits decreases in magnitude and spatial extent suggesting declining inhibitory control

**DOI:** 10.1016/j.neuroimage.2022.119081

**Published:** 2022-06

**Authors:** Stephen D. Mayhew, Sebastian C. Coleman, Karen J. Mullinger, Cam Can

**Affiliations:** a Center for Human Brain Health (CHBH), School of Psychology, University of Birmingham, Birmingham B15 2TT, United Kingdom; b Sir Peter Mansfield Imaging Center (SPMIC), School of Physics and Astronomy, University of Nottingham, Nottingham, United Kingdom; c Department of Clinical Neurosciences, University of Cambridge, Cambridge, United Kingdom

**Keywords:** Negative BOLD response, NBR, FMRI, Sensorimotor, Deactivation, Inhibition, HRF, Lifespan, Aging

## Abstract

Ipsilateral sensorimotor (iSM1) cortex negative BOLD responses (NBR) are observed to unilateral tasks and are thought to reflect a functionally relevant component of sensorimotor inhibition. Evidence suggests that sensorimotor inhibitory mechanisms degrade with age, along with aspects of motor ability and dexterity. However, understanding of age-related changes to NBR is restricted by limited comparisons between young vs old adults groups with relatively small samples sizes. Here we analysed a BOLD fMRI dataset (obtained from the CamCAN repository) of 581 healthy subjects, gender-balanced, sampled from the whole adult lifespan performing a motor response task to an audiovisual stimulus. We aimed to investigate how sensorimotor and default-mode NBR characteristics of magnitude, spatial extent and response shape alter at every decade of the aging process. We observed a linear decrease in iSM1 NBR magnitude across the whole lifespan, whereas the contralateral sensorimotor (cSM1) PBR magnitude was unchanged. An age-related decrease in the spatial extent of NBR and an increase in the ipsilateral positive BOLD response (PBR) was observed. This occurred alongside an increasing negative correlation between subject's iSM1 NBR and cSM1 PBR magnitude, reflecting a change in the balance between cortical excitation and inhibition. Conventional GLM analysis, using a canonical haemodynamic response (HR) function, showed disappearance of iSM1 NBR in subjects over 50 years of age. However, a deconvolution analysis showed that the shape of the iSM1 HR altered throughout the lifespan, with significantly delayed time-to-peak and decreased magnitude. The most significant decreases in iSM1 HR magnitude occurred in older age (>60 years) but the first changes in HR shape and timing occurred as early as 30 years, suggesting the possibility of separate mechanisms underlying these alterations. Reanalysis using data-driven HRs for each decade detected significant sensorimotor NBR into late older age, showing the importance of taking changes in HR morphology into account in fMRI aging studies. These results may reflect fMRI measures of the age-related decreases in transcollosal inhibition exerted upon ipsilateral sensorimotor cortex and alterations to the excitatory-inhibitory balance in the sensorimotor network.

## Highlights

• We studied changes to ipsilateral sensorimotor (iSM1) NBR across the adult lifespan.
• iSM1 NBR linearly decreased in magnitude and spatial extent across the whole lifespan.
• iSM1 NBR haemodynamic response shape was significantly altered from 30 years onwards.
• Canonical GLM analysis substantially under-estimated NBR in middle and old age.
• Aging increased PBR spatial extent bilaterally and the between hemisphere correlation of PBR-NBR.

## 1 Introduction

Functional magnetic resonance imaging (fMRI) is a very commonly applied technique for studying the magnitude and spatial location of neuronal activation in the brain during task performance and investigating how this activity supports cognition. fMRI infers changes in neuronal activity via fluctuations in the blood oxygenation-level dependent (BOLD) signal (Kwong et al., 1992; Ogawa et al., 1990). The BOLD signal is considered a haemodynamic signature of neural activity, it is a complex physiological response that depends upon changes in cerebral blood flow, blood volume and metabolic rate of oxygen consumption (Buxton and Frank, 1997; Buxton et al., 1998; Hoge et al., 1999; Iadecola, 2004). BOLD signals can be confounded by any factor that alters vascular reactivity or the neurovascular coupling between neural activity and the haemodynamic response (Ekstrom, 2010; Hillman, 2014; Howarth, 2014). Therefore interpreting how changes in BOLD activity reflect underlying neuronal activity is challenging, especially when studying alterations in brain function throughout the lifespan. It is crucial to disentangle age-related changes in neural activity from age-related changes in physiology in order to accurately interpret age-related changes in BOLD signal (D'Esposito et al., 2003; Tsvetanov et al., 2021).

The effects of healthy aging upon both cerebrovascular function and task-induced increases in BOLD signal, often referred to as activations, or positive BOLD responses (PBR) have been widely studied. Age-related alterations in cerebral metabolic rate of oxygen consumption (CMRO_2_) and decreases in vascular reactivity as well as both baseline and task-evoked cerebral blood flow have been observed (Ances et al., 2009; Hutchison et al., 2013a, 2013b; Liu et al., 2013; Lu et al., 2011). Further studies have shown that accounting for within- and between-subject variability in physiological factors improves assessment of changes in BOLD activation across the lifespan (Kannurpatti et al., 2014; Liu et al., 2013; Tsvetanov et al., 2015).

However, an important aspect that has received little detailed study is age-related changes in task-induced decreases in BOLD signal from baseline levels, often referred to as negative BOLD responses (NBR). NBR occur in unstimulated sensory cortex such as ipsilateral to unilateral hand movements or somatosensory stimuli (Allison et al., 2000; Hlushchuk and Hari, 2006; Kastrup et al., 2008; Klingner et al., 2011a, 2010, 2011b; McGregor et al., 2015; Mullinger et al., 2014; Newton et al., 2005; Wilson et al., 2019); or in the default mode network (DMN) during externally-engaging cognitive and sensory tasks (Buckner et al., 2008; Raichle et al., 2001). Sensory NBR are thought to represent a suppression of cortical activity in regions unnecessary for task performance to maintain efficient processing (Hairston et al., 2008; Kastrup et al., 2008; Smith et al., 2006). A wide variety of evidence suggests that NBR arise, at least in part, from a neuro-metabolic component and therefore are of interest as a marker of functional inhibition (Boorman et al., 2015; Mullinger et al., 2017, 2014; Pasley et al., 2007; Schafer et al., 2012; Shmuel et al., 2006, 2002; Sten et al., 2017). For clarity, when using the term inhibition in this paper we refer to a net suppression of excitatory neural activity such as can occur via a decrease in afferent excitation alone, or via increased local inhibition in a small number of inhibitory neurons which modulate the activity of a much larger population of excitatory neurons (Lauritzen et al., 2012; Mangia et al., 2009) and as such result in a net reduction in energy demands and a NBR.

A decrease in ipsilateral motor cortex NBR magnitude between young and old adult groups during finger movement tasks is reported (Ward and Frackowiak, 2003; Ward et al., 2008), and associated with increased extent of ipsilateral PBR (Naccarato et al., 2006; Riecker et al., 2006) Groschel et al. (2013). compared PBR and NBR to unilateral electrical median nerve stimulation between 14 young (mean age = 23 years) and 13 older (mean age = 73 years) healthy adult subjects. They reported that the significant NBR observed in ipsilateral somatosensory cortex in young subjects was absent in the old group, whilst the contralateral PBR remained strong (Groschel et al., 2013). Similarly, McGregor and colleagues studied 12 young (mean age = 22 years) and 6 older (mean age = 71 years) healthy adults performing right-hand button press tasks during fMRI at 3T (McGregor et al., 2009). They reported that the majority of young adults exhibited ipsilateral motor cortex NBR with contralateral PBR, but most of the older adults showed bilateral PBR with only one showing ipsilateral NBR. Two follow-up fMRI studies investigated the effects of fitness and showed that ipsilateral NBR was still observed in active middle-aged and older groups, although reduced compared to young adults, whereas sedentary middle-aged and older adults showed ipsilateral PBR instead (McGregor et al., 2013, 2011). Overall, these findings suggest an age-related alteration in the hemispheric balance of sensorimotor cortex activity, but also that NBR may persist into older age and display considerable between-subject variability, which requires further investigation in a larger sample.

Previous aging NBR studies are restricted by cross-sectional designs with small samples sizes and lacking study of middle (40–60 years) or late old age (>80 years) groups. Additionally, changes in NBR magnitude have been primarily measured by statistical activation mapping using the general linear model (GLM). Such analyses assume a canonical shape of the NBR haemodynamic response function (HRF) in old age (Groschel et al., 2013; Naccarato et al., 2006; Ward and Frackowiak, 2003; Ward et al., 2008). However, as age-related changes in the HRF of the PBR have been reported (West et al., 2019), it is important to investigate potential age-related changes to the shape of the NBR and their consequent implications for GLM estimation of NBR. A more comprehensive study of how the magnitude, spatial extent and shape of ipsilateral NBR and DMN NBR alter across the lifespan is needed to fully understand what aspects of the NBR are most affected by aging and the time points at which alterations emerge. Such knowledge will benefit the ability to study and interpret alterations in the NBR as a marker of changes to functional inhibition that occur in the brain during aging (Brodoehl et al., 2013) as well as during disease pathology (Butefisch et al., 2008; Manson et al., 2008).

Therefore, the aim of the current study was to improve understanding of age-related changes in NBR across the whole adult lifespan, thereby extending previous work that has largely focussed on comparing young with old samples. Here a large dataset of 581 adults who responded to a visual-auditory stimulus with a right index finger button press was used. This task generated NBR in both ipsilateral sensorimotor cortex and the DMN, thus allowing investigation of two types of NBR and comparison with sensory cortex PBR. We expected to observe age-related decreases in NBR magnitude but were motivated to examine the ages at which this decrease first appeared and how it manifested throughout the lifespan. In addition, we investigated how both the spatial extent and the shape of the NBR (i.e. the morphology of the HR timecourse) were affected by age to provide a comprehensive assessment of lifespan changes.

## 2 Materials and methods

### 2.1 Sample

All data used in the preparation of this work were obtained from the Cambridge center Aging and Neuroscience data set (Cam-Can repository, available at http://www.mrc-cbu.cam.ac.uk/datasets/camcan/) see (Shafto et al., 2014; Taylor et al., 2017) for full procedural details of the study. Ethical approval was granted by Cambridgeshire 2 Research Ethics Committee. All participants gave written informed consent. A detailed description of exclusion criteria can be found in Shafto et al. (2014). Code used to process data and prepare figures is available on request from the corresponding author.

A total of 645 healthy adult subjects were available. Out of these 581 right-handed subjects were selected based on an Edinburgh handedness score > 60 indicating right hand dominance. The age range of these subjects was 18–88 years, median = 55 years, mean = 54.6 ± 18.6 years, 291 males and 290 females. A histogram showing the distribution of subject's age is plotted in Fig. S1. To investigate progressive lifespan effects the subjects were subdivided into seven, equally-sized (*N* = 55), decile groups: Twenties, Thirties, Forties, Fifties, Sixties, Seventies and Eighties. Subjects were selected to ensure the maximum decile group size whilst ensuring an equal number of subjects in each group; to minimize overlap and provide age distinction between groups; to balance gender as well as possible within each decile group. See Table 1 for demographic information of each decile group.

**Table 1:** Demographic information, MMSE and ACE-R scores, behavioral performance during the fMRI sensorimotor task (SMT) and framewise displacement (FD) summary of head motion. Each measure was averaged across participants in each of the seven decile groups used in this study. Aside from age-range and gender all measures are reported as mean ± standard deviation.

	**Twenties**	**Thirties**	**Forties**	**Fifties**	**Sixties**	**Seventies**	**Eighties**
**Age (years)**	25.8 ± 2.4	34.7 ± 1.7	44.9 ± 1.7	54.1 ± 2.0	64.2 ± 1.9	73.9 ± 2.1	82.6 ± 2.3
**Age range**	20–29	32–38	42–48	52–58	61–68	71–78	80–88
**Gender (M,F)**	27,28	28,27	26,29	29,26	27,28	27,28	28,27
**Handedness**	92.5 ± 11.4	94.3 ± 8.9	92.5 ± 11.2	94.9 ± 8.8	96.0 ± 7.5	96.7 ± 6.2	96.5 ± 6.5
**MMSE**	29.2 ± 1.3	29.1 ± 1.3	29.2 ± 1.0	29.2 ± 1.1	28.8 ± 1.2	28.1 ± 1.5	28.0 ± 1.5
**ACE-R**	95.4 ± 4.8	97.2 ± 3.3	96.9 ± 3.1	95.6 ± 3.6	95.2 ± 3.8	93.1 ± 4.4	90.1 ± 7.0
**SMT accuracy (%)**	98.9 ± 6.1	99.9 ± 0.2	99.8 ± 0.5	99.4 ± 2.6	98.1 ± 5.1	98.5 ± 5.7	99.0 ± 3.6
**SMT RT (ms)**	300 ± 68	302 ± 59	328 ± 84	308 ± 72	320 ± 70	302 ± 71	301 ± 79
FD	63.5 ± 28.0	62.1 ± 25.7	79.5 ± 39.7	86.7 ± 33.9	93.9 ± 36.7	130.3 ± 73.4	135.5 ± 61.5

### 2.2 Data acquisition - paradigms

fMRI data were acquired whilst participants performed a simple audiovisual sensorimotor task (SMT). In the sensory task, participants responded to a total of 129 trials which consisted of an initial practice trial, 120 bimodal audio/visual trials, and eight unimodal trials included to discourage strategic responding to one modality (four visual only and four auditory only). The timing of trials were optimized for estimation of the fMRI impulse response by generating a sequence of stimulation and null trials using a 255-length m-sequence (Buracas and Boynton, 2002) with *m* = 2 and minimal stimulus onset asynchrony (SOA) of 2 s (resulting in SOAs ranging from 2 to 26 s). For each bimodal trial, participants saw two checkerboards presented bilaterally for 34 ms, to the left and right of a central fixation, and simultaneously heard a 300 ms binaural tone at one of three frequencies (300, 600, or 1200 Hz, equal numbers of trials pseudorandomly ordered). For unimodal trials, participants either only heard a tone or saw the checkerboards. For each trial, participants responded by pressing a button with their right index finger if they heard or saw any stimuli (Shafto et al., 2014).

Subjects also completed a battery of cognitive and behavioral assessments. Measures from the Edinburgh Handedness Inventory (Oldfield, 1971) were used to identify right-handed subjects. The current study also used the Mini-Mental State Examination (MMSE; (Folstein et al., 1975)) and the Addenbrooke's Cognitive Examination (ACE-R; (Mioshi et al., 2006)) scores to evaluate cognitive decline in the sample. We also used additional behavioral measures that had relevance to sensorimotor function and studied their correlation with age and potential functional relationship with BOLD responses. In addition to reaction time of the button press to the audiovisua stimulus during the MRI scan (mRT), we also examined data from the following four tasks conducted outside the scanner on a separate visit: a simple reaction time (sRT) assessment using right index button press to visual instruction, a choice reaction time (cRT) task using right index button press to visual instruction, motor learning task (ML, horizontal stylus movements to hit targets, before, during and after manipulations of the cursor position relative to the stylus), and a force matching task (FM, using right index finger to either directly or indirectly match a force applied to left index finger). For full details of these tasks see (Shafto et al., 2014). Linear regression against age and one-way ANOVA were used to check for age-related effects in these measures across deciles and relation to BOLD responses measured as outlined below.

### 2.3 Data acquisition - MRI

MR data were acquired using a 3T Siemens TIM Trio using a 32 channel head coil at the MRC—Cognition and Brain Sciences Unit (CBSU), Cambridge University. T2*-weighted fMRI data were acquired using a gradient-echo echo-planar imaging sequence with 32 axial slices (acquired in descending order), slice thickness was 3.7 mm with an interslice gap of 20% (for whole brain coverage including cerebellum), leading to a voxel-size = 3 mm × 3 mm × 4.44 mm; TR = 1970 ms; TE = 30 ms; flip angle = 78°; FOV = 192 mm × 192 mm. A total of 261 volumes were acquired with an acquisition time of 8 min and 40 s. A spoiled gradient-recalled (SPGR) gradient-echo sequence with the same parameters as the fMRI data was acquired, but with two TEs (5.19 ms and 7.65 ms). The acquisition time was 54 s. The phase difference between the two TEs was used to calculate field maps in order to unwarp BOLD image distortions caused by magnetic field inhomogeneities surrounding tissue, bone and air interfaces in the head. A high resolution 3D T1-weighted structural image was acquired using an MPRAGE sequence with the following parameters: TR = 2250 ms; TE = 2.99 ms; TI = 900 ms; flip angle = 9°; FOV = 256 mm x 240 mm x 192 mm; voxel size = 1 mm isotropic; GRAPPA acceleration factor = 2. Finally, a high-resolution 3D T2-weighted structural image was acquired with a SPACE sequence with the following parameters: TR = 2800 ms; TE = 408 ms; FOV = 256 mm × 256 mm × 192 mm; resolution = 1 mm isotropic; GRAPPA acceleration factor = 2 (Shafto et al., 2014).

### 2.4 Data processing and analysis

Each participant's T1 image was coregistered to the MNI template in SPM12 (http://www.fil.ion.ucl.ac.uk/spm), and the T2 image was then coregistered to the T1 image using a rigid-body transformation. The coregistered T1 and T2 images underwent multi-channel segmentation (SPM12 Segment; (Ashburner and Friston, 2005)) to extract probabilistic maps of six tissue classes: GM, WM, cerebrospinal fluid (CSF), bone, soft tissue, and background. The native-space GM and WM segmentations were used for diffeomorphic registration (DARTEL; (Ashburner, 2007)) to create whole group template images (Taylor et al., 2017). The group template was normalized to the MNI space using 12-parameter affine transformation.

The fMRI data were unwarped using the field-maps to compensate for magnetic field inhomogeneities, realigned to correct for head motion, and slice-time corrected to the middle slice. The normalization parameters from the T1 image processing were then applied to warp functional images into MNI space. A data-driven wavelet despiking was applied to minimize motion artefacts (Patel et al., 2014), which has been demonstrated to capture a large amount of motion artefacts in the data (Tsvetanov et al., 2020).

GLM analysis of the sensory fMRI data was conducted in FEAT 6.00 (https://fsl.fmrib.ox.ac.uk/fsl/). Data were spatially smoothed with a 5 mm FWHM Gaussian kernel and FILM prewhitening was applied (Woolrich et al., 2001). At the first-level, the five conditions (audio alone, visual alone, visualaudio300Hz, visualaudio600Hz, visualaudio1200Hz) of the sensory task were modeled with five separate regressors (EVs). 50 ms duration delta functions were used to represent each event based on the stimulus timings. All stimulus regressors were convolved with the canonical double gamma HRF. Only trials with correct button press response were modeled, group mean overall task accuracy was 99.0 ± 0.03% reflecting very high performance and very few trials omitted. The six realignment parameters from the motion correction were also included in the design matrix as regressors of no interest. Contrasts were used to calculate the average response across all five conditions and both positive and negative contrasts were used to estimate the mean PBR and mean NBR for each subject. In this work all analyses refer to the contrasts that combined data across all conditions, as differences between conditions were not of interest here.

A series of second-level, group analyses were then performed to investigate age-related effects on PBR and NBR. All higher level analyses were computed in FEAT using mixed effects FLAME 1 + 2 (Woolrich et al., 2009), with a Z-statistic threshold > 3.1 and cluster corrected at *p*<0.05. Firstly, a second-level GLM was constructed that calculated the mean PBR and mean NBR for each of the seven decile groups described above, and separately tested for significant differences in PBR or NBR between each pair of decile groups. This analysis would enable inspection of PBR and NBR in each decade of the lifespan and investigation of their changes with age. Secondly, two additional second-level GLMs were constructed using all 581 subjects that included two regressors: the value of the first regressor was set to one for each subject to model the group mean response; the second regressor contained either subject's age or handedness (mean subtracted) in order to model between-subject variability in those metrics. Positive and negative contrasts were set on both of the regressors; statistical maps from regressor one represented group mean PBR and NBR; statistical maps from regressor two showed the brain areas with a positive or negative correlation between the fMRI response and subject's age or handedness. Regions of interest (ROIs) were defined to facilitate further analysis of aging effects. The following regions were identified to study PBRs: contralateral sensorimotor cortex (cSM1), primary visual (V1) cortex and bilateral thalamus. The following regions were identified to study NBRs: ipsilateral sensorimotor cortex (iSM1) and the posterior cingulate cortex (PCC) of the default mode network (DMN). These regions were selected to encompass the key cortical and subcortical regions involved in processing the task. In all cases, ROIs were defined from masking the group mean PBR and NBR maps with the respective mask from the FindLab atlas (https://findlab.stanford.edu/functional_ROIs.html) (Shirer et al., 2012). For each anatomical ROI the peak response voxel was located from the first level data for each subject and a cubic ROI (3 × 3 × 3 = 27 voxels) was centered on that peak voxel, to give a subject specific functional ROI for each of the anatomical regions. Mean Z-statistic values, beta-weight (the GLM regression coefficient), values were extracted for each functional ROI for each subject as a measure of the peak response. The mean BOLD signal timecourse from each of the ROIs was also extracted. In addition, for both PBR and NBR and in each decile, the ROIs were used to obtain the peak voxel Z-statistic and MNI co-ordinate both from: the second-level group maps; and the average of the first-level subject maps.

To investigate head motion in the data and its potential relationship with age and BOLD responses we used the six realignment parameters from the motion correction for each subject to calculate the framewise displacement (FD) (Power et al., 2012) which indexes how much the head moves from volume to volume. For each subject, FD values were summed across volumes to assess total motion. We then used one-way ANOVA to test for any effect of decile upon FD, and Pearson's correlation to investigate relationship between FD and: age, cSM1 PBR and iSM1 NBR.

For each ROI the linear Pearson correlation between beta-weight and age was calculated, to enable clear visualization of the shape of that relationship. Furthermore, the linear Pearson correlation between cSM1 PBR and iSM1 NBR was calculated for each decile group as well as across all 581 subjects. This enabled investigation of potential relationships between the primary excitatory and inhibitory responses within the sensorimotor system and whether they remained consistent across the lifespan.

To assess the spatial extent of the PBR and NBRs in the sensorimotor cortex, the number of voxels with a positive or negative, beta-weight in the bilateral sensorimotor cortex mask was calculated from each subject's first-level data. Beta-weights were thresholded to remove the effects of noisy voxels with values close to zero. For each subject, and separately for positive and negative responses, only beta-weights larger than 5% of the subject's maximum value were used. For each subject the number of PBR and NBR voxels were both converted into a proportion of the size of the sensorimotor cortex mask and the ratio between PBR/NBR calculated. One way ANOVA were used to test for significant effects of decile group on these metrics so that we assessed how the spatial extent of PBR and NBR in the whole sensorimotor cortex, and the ratio between them, changed across the lifespan. This enabled exploration of whether age-related changes in the extent of NBR occurred alongside changes to the extent PBR, or whether changes were specific only to the NBR.

To investigate aging effects on the morphology of the PBR and NBR, BOLD haemodynamic response (HR) timecourses from each ROI were obtained using the deconvolution method of Lu et al. (2006). Analysis of fMRI data has been long founded on the assumed relationship between neural activity and the subsequent haemodynamic BOLD response, which is typically described as:

(1) HR=neuralinput*HRF

where: HR is the evoked haemodynamic response, HRF is the haemodynamic impulse response function and * denotes the convolution of the two functions (Boynton et al., 1996). The HRF translates known neural activity into a predicted BOLD response. To truly estimate the underlying HRF one needs to either measure the neural input or manipulate the neural input (e.g. by varying the stimulus duration) such that a constant HRF can be estimated that fits multiple duration inputs (Grinband et al., 2017). Neither of these options were available for this dataset, therefore we did not assume a specific neural input and our deconvolution analysis used the button press timings as a constant amplitude delta function (as were input to the original GLM). This enabled us to estimate the specific HR to this particular stimulus for each subject, but not the more general HRF. This deconvolved signal is the result of neural input of unknown magnitude and duration, and an unknown HRF, any of which may change with age. We aim to make inferences about aging effects on the neural input by comparing BOLD responses, taken from spatially distinct regions with subjects, across the lifespan. In doing so we assume that vascular compliance, which will affect the HRF, is consistent between regions.

Using deconvolution HRs were extracted from each ROI for each subject. The peak magnitude and time-to-peak of each subject's HR were measured from the maximal signal change, separately for each ROI. Finally, for each ROI one-way ANOVA were used to test for significant effects of decile upon these HR parameters. Subsequent post-hoc t-tests were used to further explore any significant effects. In addition the HRs for each ROI were averaged within each of the decile groups to enable visualization of the entire HR shape for each region.

As is described fully in the results, we found that the magnitude and the shape of the HR derived from iSM1 altered significantly with age. Therefore, we performed further analysis to investigate the extent to which deviations in NBR shape from using a canonical HRF could explain lifespan changes in NBR activation maps calculated using the GLM. First-level GLM analyses were recalculated using the mean iSM1 HR from each subject's decile to perform the convolution of event timings when forming the design matrix, e.g. the mean iSM1 HR from the Eighties decile (red line in Fig. 9D) was used as the convolution kernel for all subjects from the Eighties decile. HRs were normalized before the convolution was applied, by dividing by the maximum amplitude, in order to preserve equivalent HR magnitude across deciles. Equivalent procedures were performed for all deciles. All other GLM analysis parameters were kept the same as the initial, conventional canonical analysis. Group level NBRs were estimated for each decile and statistical maps and beta-weights for each ROI were compared between the canonical HRF and data-driven HR analyses. Here we aim to evaluate whether changes in NBR with age are due to lifespan alterations in HR shape, which confound the canonical GLM fit, as well as changes in response magnitude. Furthermore, running this second GLM provides additional information about changes in the spatial extent of the NBR with age.

## 3 Results

### 3.1 Lifespan changes in cognition and behavior

One-way ANOVA found a significant difference in MMSE (F(1,6)=8.8, *p* = 5 × 10^−9^) and ACE-R (F(1,6)=14.3, *p* = 1 × 10^−15^) but no difference in either accuracy (F(1,6)=1.5, *p* = 0.33) or RT (F(1,6)=1.3, *p* = 0.27) in the SMT across deciles, Table 1. For MMSE and ACE-R, post-hoc t-tests showed that this difference arose primarily due to decreases in older age, with significant differences between the Seventies and Eighties deciles and all of the other deciles (*p*<0.05). One-way ANOVA also found a significant difference in Handedness across deciles (F(1,6)=3.3, *p*<0.01). Post hoc t-tests revealed significant differences between the Twenties decile and the Sixties (*p*<0.02), Seventies (*p*<0.002) and Eighties (*p* = 0.005) deciles; as well as between the Forties decile and both the Seventies (*p*<0.005) and Eighties (*p* = 0.02) deciles. In all these cases, older subjects were more right-handed than the younger subjects. MMSE (*R*=−0.31, *p* = 6 × 10^−14^) and ACER (*R*=−0.36, *p* = 1 × 10^−19^) scores were significantly and strongly negatively correlated with age and handedness was significantly positively correlated (*R* = 0.15, *p* = 4 × 10^−5^) with age.

Subject's mRT (during the fMRI task) showed no relationship with age (*R* = 0.001, *p* = 0.98). In contrast, both sRT (*R* = 0.37, *p* = 6 × 10^^−19^) and cRT (*R* = 0.62, *p* = 5 × 10^−59^) showed significant and robust positive correlations with age, indicating that RT increased in older age. At each of the five phases of the motor learning task a significant positive correlation between age and the subject's time taken to hit the target (*R* = 0.46–0.55, all *p*<10^^−15^) was observed. In the force matching task, significant positive correlations were observed between age and force overcompensation in the direct condition (*R* = 0.27, *p* = 6 × 10^−6^) and reaction time in the indirect condition (*R* = 0.29, *p* = 7 × 10^−7^).

### 3.2 fMRI results

Mean FD values, summarizing head motion per decile, are displayed in Table 1. We found a significant increase in FD across deciles (one way ANOVA) F(18.6), *p*<1 × 10^−14^. This result was accompanied by a significant positive, between-subjects, correlation of FD with age, *R* = 0.49, *p* = 1 × 10^−10^. However, no correlation was observed between FD and the GLM beta-weights from either the cSM1 PBR (*R* = 0.005, *p* = 0.90) or iSM1 NBR (*R*=−0.02, *p* = 0.61) ROIs. These results suggest that head motion increases with age, similar to previous reports (Madan, 2018; Savalia et al., 2017), but was not related to PBR and NBR magnitude in this study.

**Fig. 1:**
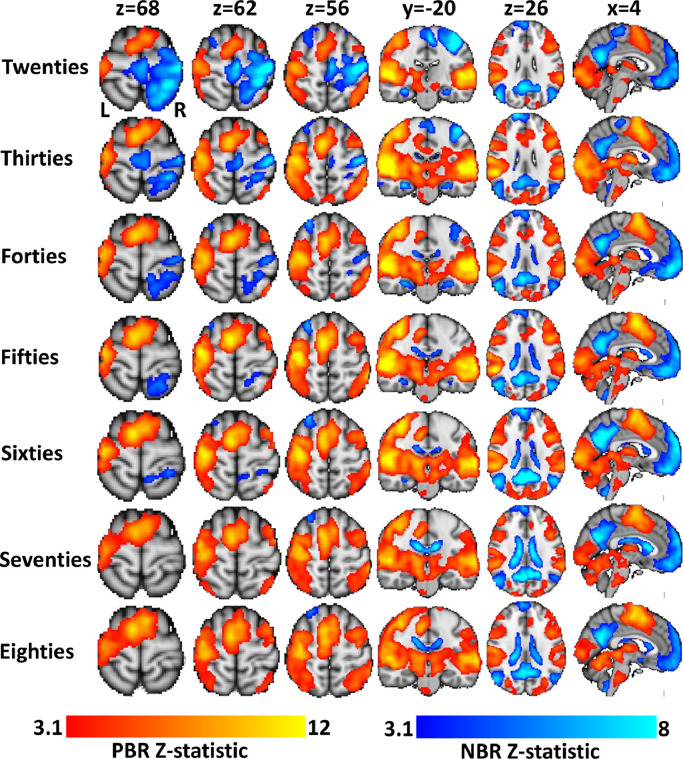
Variation in main-effect BOLD task responses across the lifespan calculated using general linear modeling with a canonical haemodynamic response function. Each row displays the mean positive (PBR, red-yellow) and negative BOLD response (NBR, blue) of each the seven decile groups (Twenties-Eighties). Columns 1–4 show axial and coronal slices through the sensorimotor cortex, illustrating how the ipsilateral sensorimotor cortex (iSM1) NBR decreases with age. Columns 5,6 show axial and sagittal slices through the center of the brain, showing the consistency in the DMN NBR across the lifespan. (For interpretation of the references to color in this figure legend, the reader is referred to the web version of this article.)

Fig. 1 shows the average main-effect PBR (red-yellow) and NBR (blue) to the sensory task for each decile group. In all deciles significant PBR was observed in contralateral sensorimotor cortex (cSM1) and the bilateral primary visual and auditory cortices that were directly stimulated by the task paradigm. PBR was also observed in the anterior midline supplementary motor cortex, anterior cingulate cortex, dorsal parietal cortex, thalamus, basal ganglia, brainstem and cerebellum.

Significant NBR was observed in ipsilateral sensorimotor cortex (iSM1) in only three of the decile groups: Twenties; Thirties and Forties. In the Fifties and Sixties, a small NBR remained in ipsilateral dorsal parietal cortex which disappeared in the Seventies and Eighties. NBR was observed in the core regions (PCC, bilateral intra parietal lobe, medial prefrontal cortex, medial temporal lobe and hippocampus) of the DMN in all decile groups. The spatial extent of the DMN NBR was very similar for all decile groups apart from the hippocampus, which showed NBR in the Twenties-Sixties deciles (see Fig 1, column 4) but no response in the Seventies and Eighties. NBR were also observed in the CSF of the ventricles, especially in the oldest deciles.

**Fig. 2:**
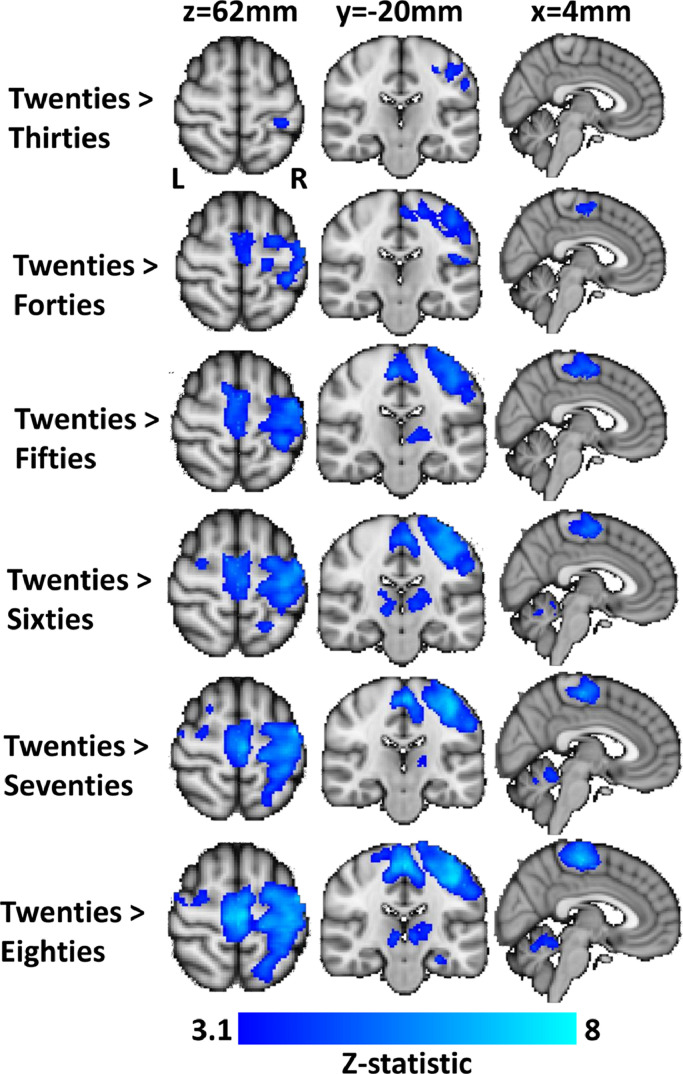
Regions of significant difference in BOLD responses between the Twenties decile and each of the other decile groups. Differences are observed primarily in regions of ipsilateral sensorimotor cortex (iSM1) and supplementary motor cortex that show a main-effect NBR on average, but also in the thalamus and cerebellum that show a PBR on average.

Significant differences in BOLD response between deciles were only observed when contrasting the Twenties with the other groups, as displayed in Fig. 2. When compared with every other decile, significantly larger NBR in iSM1 was observed in the Twenties decile. This difference was restricted to a focal area of iSM1 for Twenties > Thirties, which expanded to encompass all of iSM1 and midline supplementary and premotor areas when comparing between Twenties and older decile groups. No differences in DMN NBR were observed in any of the contrasts, supporting Fig. 1 which showed comparable DMN NBR at all ages. Significant differences between deciles were also observed in regions of the thalamus that showed a main effect PBR on average. No differences in the BOLD response were observed between decile groups in visual, auditory or cSM1 primary sensory cortex regions, suggesting that the PBR did not change substantially across the lifespan.

**Fig. 3:**
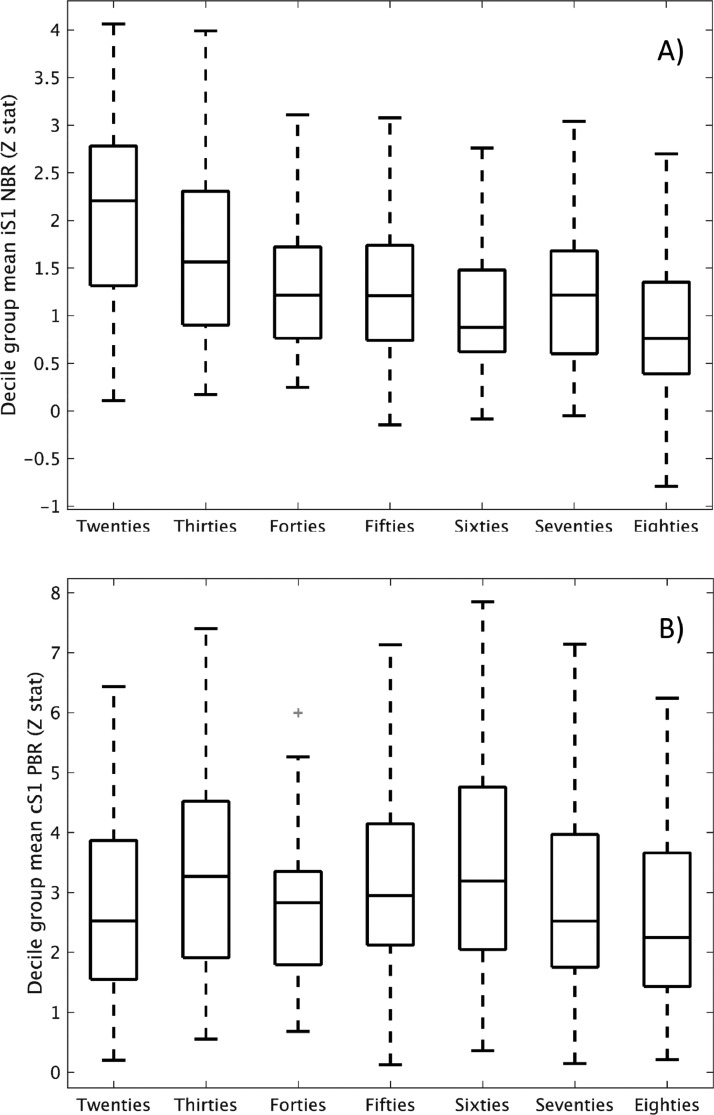
Box plots of the mean of the first-level iSM1 NBR ROI (A) and cSM1 PBR ROI (B) peak Z-statistic for each decile group, showing the mean, interquartile range and maximum and minimum of the data. Black cross indicate outlying subjects.

Mean first-level Z-statistics extracted from the iSM1 ROI are displayed as boxplots per decile in Fig. 3A to illustrate their distributions. Young adults, particularly in their Twenties, showed an appreciably stronger NBR than was seen over the rest of the lifespan. This data suggests that the NBR doesn't entirely disappear, as the group GLM result (Fig. 1) indicated, but is still present to a small extent across the rest of lifespan as the mean and interquartile range are above zero for all deciles. Boxplots for the deciles Forties-Eighties show highly comparable mean, range and variance. The equivalent boxplots for the cSM1 PBR show the similarity in that response across deciles (Fig. 3B) Table 2.A provides further data on the consistency of cSM1 PBR and changes in iSM1 NBR across the lifespan of the peak voxel Z-statistic (both group and first level) and peak voxel co-ordinates. Analysis of the iSM1 ROI at the first-level, expressed as a percentage of the Twenties value, shows that the mean Z-statistic decreases to 80%, 63%, 59%, 51%, 57% and 41% across the other six deciles; whereas the cSM1 ROI Z-statistic was comparatively consistent at 108%, 96%, 113%, 109%, 97% and 95% across the other six deciles.

**Table 2:** Summary of consistency of contralateral sensorimotor cortex (cSM1) positive BOLD response (PBR) and changes in ipsilateral sensorimotor cortex (iSM1) negative BOLD response (NBR) across the lifespan. Displays data measured from second-level, group maps (peak voxel co-ordinate, peak Z-statistic) as well as 1st level measures (mean ROI Z-statistic,% of Twenties) for two sets of GLM results: (A) using the canonical HRF (first 8 rows) as shown in Fig. 1; and (B) the data derived HR (second 8 rows) as shown in Fig. 10. Data for each decile are shown in individual columns.

	Twenties	Thirties	Forties	Fifties	Sixties	Seventies	Eighties
***A. Canonical HRF***							
**cSM1 Group level peak voxel coord**	42,−20,54	42,−18,56	40,−20,56	40,−16,58	38,−22,58	38,−26,60	38,−22,58
**cSM1 Group level peak Z-stat**	9.39	11.6	11.2	12.9	12.0	10.5	10.33
**cSM1 mean 1st level Z-stat**	2.87 ± 1.69	3.11 ± 1.71	2.76 ± 1.77	3.24 ± 1.84	3.12 ± 1.66	2.78 ± 1.65	2.74 ± 1.76
**cSM1 mean 1st level Z-stat (%Twenties)**	100%	108%	96%	113%	109%	97%	95%
**iSM1 Group level peak voxel coord**	−40,−18,60	−40,−18,60	−40,−18,60	–	–	–	–
**iSM1 Group level peak Z-stat**	10.8	10.5	6.1	0	0	0	0
**iSM1 mean 1st level Z-stat**	2.11 ± 0.98	1.67 ± 0.90	1.33 ± 0.74	1.24 ± 0.79	1.07 ± 0.70	1.21 ± 0.72	0.85 ± 0.77
**iSM1 mean 1st level Z-stat (%Twenties)**	100%	80%	63%	59%	51%	57%	41%
***B. Data driven HRF***							
**iSM1 Group level peak voxel coord**	−42,−22,58	−40,−22,62	−34,−24,64	−36,−26,66	−24,−26,70	−24,−26,70	−24,−24,70
**iSM1 Group level peak Z-stat**	10.6	9.9	10.2	8.5	9.1	5.9	5.8
**iSM1 mean 1st level Z-stat**	2.15 ± 1.03	2.03 ± 1.00	1.94 ± 1.02	1.75 ± 1.04	1.75 ± 0.95	1.58 ± 0.81	1.38 ± 0.91
**iSM1 mean 1st level Z-stat (%Twenties)**	100%	95%	90%	81%	81%	74%	64%

These results suggest that the iSM1 NBR declines from an early age, decreases are observed within young adults that become highly pronounced by middle and older age to the extent that iSM1 NBR disappears from the group GLM maps by the time subjects reach their fifties. The decline in iSM1 NBR appears to be not just associated with old age, but with healthy aging across the lifespan. In contrast, the DMN NBR to this task appears unaffected by age.

**Fig. 4:**
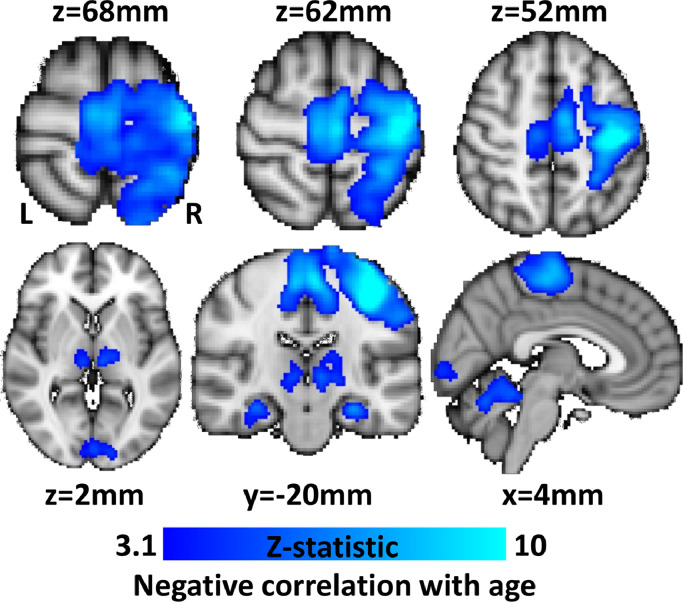
Maps of the group-level covariation of the BOLD response with age as the between subject regressor (EV). Significant negative correlation was observed between subject's age and the BOLD response to the task primarily in NBR regions of sensorimotor cortex. Correlations were also observed in thalamus, primary visual cortex and cerebellum.

Fig. 4 shows the results of using the GLM to test for correlations between the variation in age of all 581 subjects and the BOLD response across the whole brain. This analysis showed that the dominant effect of age was on the NBR in iSM1. A significant negative correlation was observed between age and NBR in iSM1, extending into ipsilateral parietal cortex, as well as in midline sensorimotor regions. Negative correlations with age were also observed in small portions of the bilateral IPL and also the bilateral hippocampus regions of the DMN. In addition, a negative correlation with age was observed in regions that showed a main effect PBR to the sensory task such as the bilateral thalamus, the anterior cerebellum and a small region of the primary visual cortex. In contrast, the group GLM using handedness as a between-subject covariate found no significant group-level correlations with either PBR or NBR, suggesting variations in the extent of right handedness were not a strong factor in modulating the BOLD response to this task, despite the subject group showing some increases in right-handedness in older age.

**Fig. 5:**
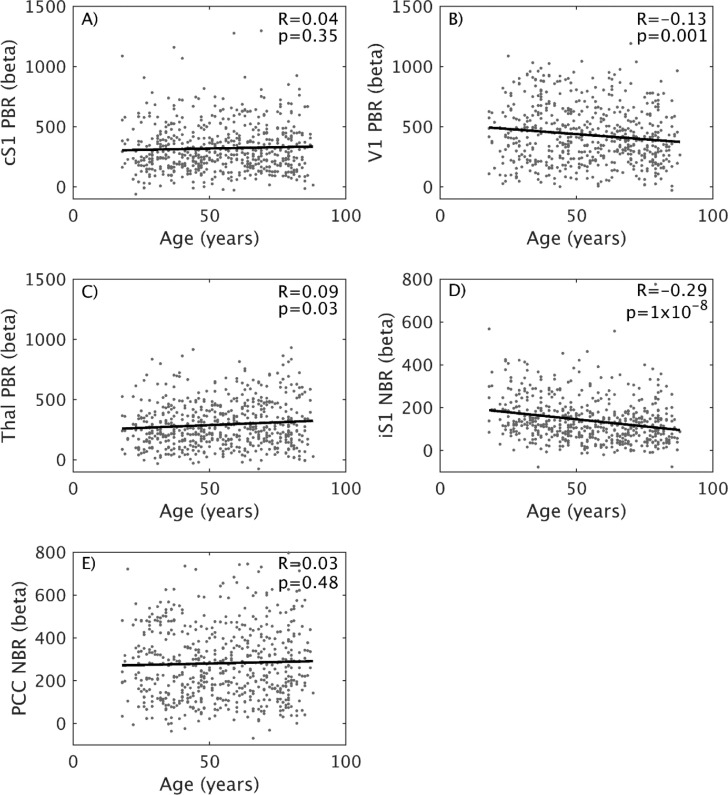
Linear correlation across all subjects (*N* = 581) between age and the peak beta-weight of four ROIs: (A) cSM1 PBR; (B) visual PBR; (C) thalamus PBR; (D) iSM1 NBR; (E) default mode network posterior cingulate cortex (DMN PCC) NBR . Additional linear fits were performed (not shown on figure) separately for youngest (<45), middle aged (45–65) and eldest (>65 years), which in the iSM1 showed steeper decline in young (*R*=−0.25) than middle (*R*=−0.12) or oldest (*R*=−0.11) age.

To further visualize the relationship between age and BOLD response Fig. 5 shows plots of the linear correlation between age and mean first-level beta-weight for each of the ROIs. No significant relationship was found for either cSM1 PBR (Fig 5A) or PCC NBR (Fig 5E) ROIs. A significant but weak (*R*=−0.13, *p* = 0.001) negative correlation with age was observed in the V1 PBR ROI (Fig 5B). A positive correlation (*R* = 0.09, *p* = 0.03) was observed in the thalamus PBR ROI (Fig 5C), but this would not be significant if the alpha level of significance was corrected to 0.01 to account for multiple comparisons (five tests). A significant (*R*=−0.29, *p*<1 × 10^−8^) strong negative correlation between age and NBR was observed in the iSM1 ROI (Fig 5D), with age explaining 15% of the variance in iSM1 NBR across the cohort. Additional linear fits were performed separately for youngest (<45), middle aged (45–65) and eldest (>65 years) subjects, which in the iSM1 ROI showed considerably steeper decline in the youngest (*R*=−0.25) than in either middle (*R*=−0.12) or oldest (*R*=−0.11) age. In addition, despite the lack of correlation of PCC NBR with age, we did observe a positive correlation (*R* = 0.26, p-1 × 10^−10^) between iSM1 NBR and PCC NBR magnitudes across the group.

**Fig. 6:**
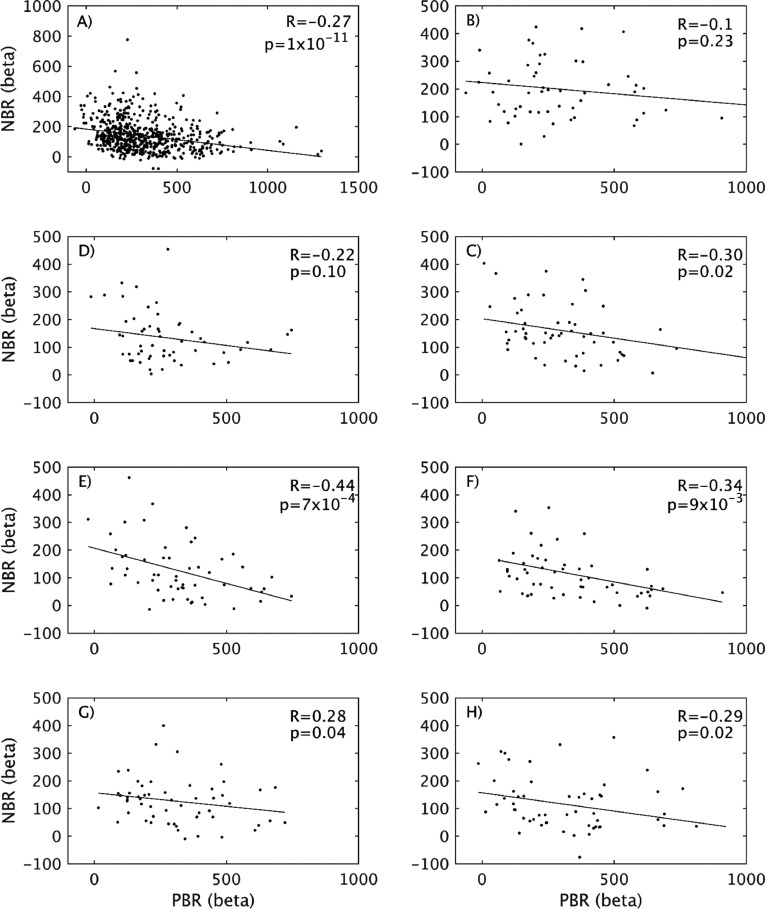
Investigation of lifespan changes in relationship between peak cSM1 PBR - iSM1 NBR magnitudes. Linear correlation between the mean first-level beta-weight of the cSM1 PBR and iSM1 NBR ROIs, shown over all 581 subjects (A) and separately for each decile group: Twenties (B), Thirties (C), Forties (D), Fifties (E), Sixties (F), Seventies (G), Eighties (H).

These analyses have clearly demonstrated that the inhibition of the ipsilateral sensorimotor cortex (reflected by iSM1 NBR) decreases across the lifespan, whilst activation of the same cortical modality (reflected by cSM1 PBR) remains relatively consistent. Therefore, we next investigated lifespan changes to the relationship between cSM1 PBR and iSM1 NBR, starting with the correlation between their beta-weight peak magnitudes. We examined each decile group as well as the relationship across the whole group. As shown in Fig. 6A, across the whole group of 581 subjects there was a significant and strong negative correlation between cSM1 PBR and iSM1 NBR magnitudes, reflecting that the largest magnitude PBR was observed in the subjects with smallest NBR (and vice versa). Inspection of this correlation in each decile showed that this relationship was not consistent across the lifespan, as the correlation was not present in the Twenties and Thirties deciles (Fig 6B-C), but only emerged with aging. The negative relationship was strongest in the Fifties and Sixties deciles (Fig 6D–F) and then weakened in the Seventies and Eighties deciles (Fig 6G-H). If Bonferroni correction for eight multiple comparisons is performed, then only the Fifties and Sixties deciles remain significant (*p*<0.006). This result is interesting as we observe the largest decreases in NBR magnitude between the Twenties and Forties (Figs. 1, 5D), which we now find is associated with the appearance of a linear correlation between cSM1 PBR and iSM1 NBR magnitude. We further investigated the extent to which this relationship is driven by age by removing the effect of age separately from both the cSM1 PBR and iSM1 NBR beta-weights. Using the whole dataset of 581 subjects, each BOLD response was separately linearly regressed against age and the residual of the fit taken as the “corrected” cSM1 PBR or iSM1 NBR responses. These were then divided into deciles and linear PBR-NBR correlations performed again. We found that the pattern of the PBR-NBR relationship was unchanged across the deciles, suggesting that age does not drive this coupling.

**Fig. 7:**
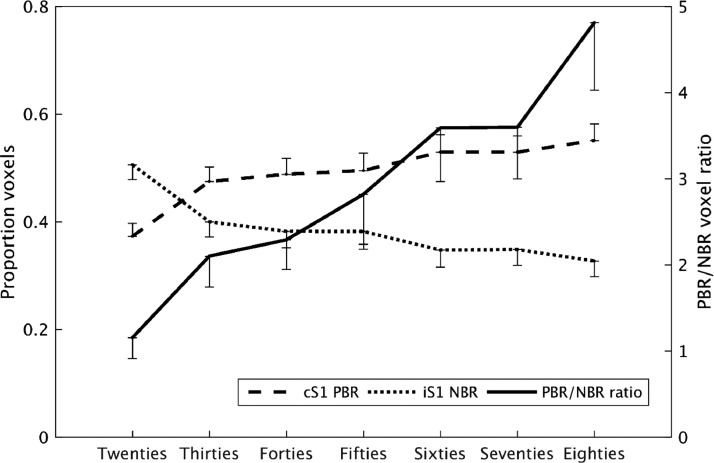
Investigation of lifespan changes in cSM1 PBR and iSM1 NBR spatial extent. The mean proportion of sensorimotor cortex voxels showing PBR (positive beta-weight) or NBR (negative beta-weight) at the first-level, and the consequent ratio of PBR/NBR, is plotted per decile group. Only beta-weights larger than 5% of the subject's maximum value were used. PBR (dashed) and NBR (dotted) proportion are plotted on the left vertical axis and PBR/NBR ratio (thick line) is plotted on the right vertical axis. Error bars denote standard error in the mean.

We next examined how the spatial extent of PBR and NBR within the whole, bilateral, sensorimotor cortex varied across the lifespan Fig. 7. plots how the proportion of sensorimotor cortex PBR (positive beta-weight) voxels and NBR (negative beta-weight) voxels changed over the lifespan. This provides further evidence that the NBR declined steadily with age, as the proportion of NBR voxels decreased in every decile. In the Twenties decile NBR comprised a larger proportion (51%) of whole sensorimotor cortex than PBR (37%), which reverses to 31% NBR and 55% PBR in the Eighties decile. The NBR decrease was mirrored by a progressive increase in the extent of PBR with age, resulting in a large increase in the ratio of PBR to NBR (thick line) in the sensorimotor cortex over the lifespan. One-way ANOVA showed a significant effect of decile on PBR proportion (F(1,6)=4.5, *p* = 2.5 × 10^−4^), NBR proportion (F(1,6)=4.4, *p* = 2.3 × 10^−4^), and PBR/NBR ratio (F(1,6)=1.9, *p* = 0.03). These results suggest that we do not simply observe only a decrease in the spatial extent and magnitude of iSM1 NBR but also a coincident increase in the spatial extent of cSM1 PBR, despite measuring no change in cSM1 PBR magnitude. This results in a greater proportion of sensorimotor cortex becoming activated, such that the relative proportion of PBR to NBR gradually increases over the lifespan.

**Fig. 8:**
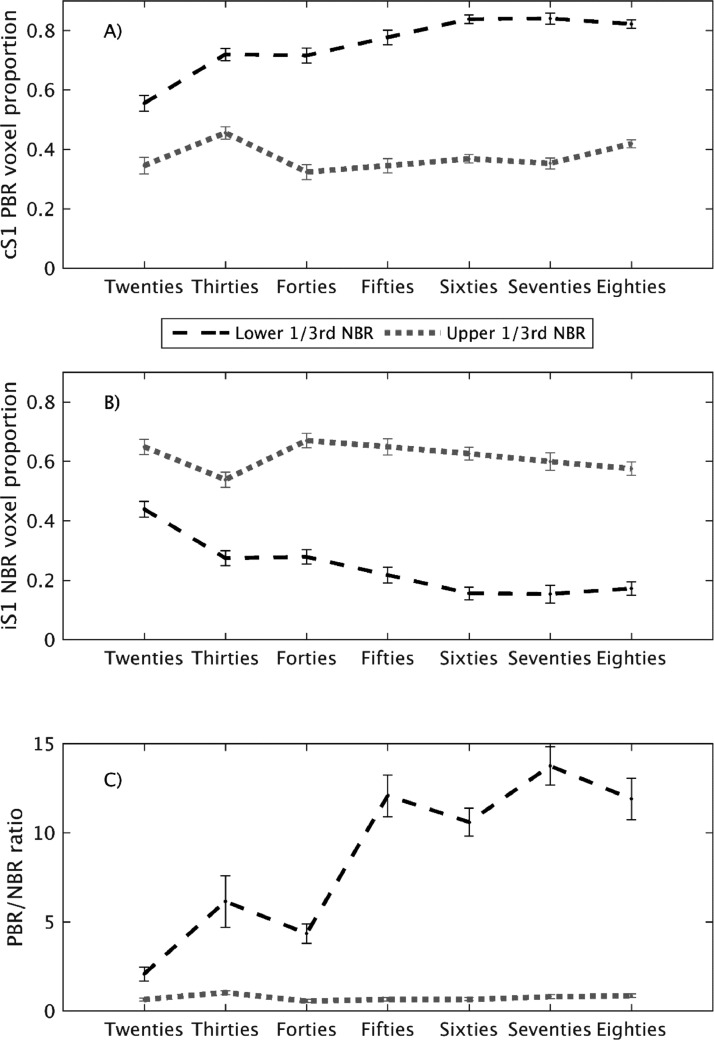
Comparison of lifespan changes in cSM1 PBR and iSM1 NBR spatial extent between the subjects with the strongest and the weakest NBR. Average cSM1 PBR (A), iSM1 NBR (B) voxel proportion and PBR/NBR ratio (C) are plotted for lower (black dashed) and upper (gray dotted) tertile NBR subjects, for each decile group. Error bars denote standard error in the mean. (For interpretation of the references to color in this figure legend, the reader is referred to the web version of this article.)

This finding is further elucidated by comparing the PBR and NBR ratio between those subjects that displayed the least and the most NBR. Each deciles subjects were divided into lower (*N* = 18), middle (*N* = 19) and upper (*N* = 18) thirds (tertiles) based on their peak iSM1 NBR Z-statistic. The equivalent analysis to Fig. 7 was then conducted for the lower and upper NBR tertile subjects in each decile age group. The results are plotted in Fig. 8 which shows that lifespan changes in PBR and NBR extent are markedly different between the lower and upper NBR tertiles. In the subjects with the most NBR (gray dotted lines), the level of PBR and NBR extent was comparable across all deciles, around approximately 60% NBR and 40% PBR. In these subjects both the PBR (One-way ANOVA, F(1,6)=1.4, *p* = 0.11) and the NBR (F(1,6)=1.3, *p* = 0.16) proportions showed no alteration with age (Fig. 8A and B), resulting in a consistent PBR/NBR ratio of approximately 0.7 (Fig. 8C) that also showed no effect of decile (F(1,6)=1.7, *p* = 0.09). This showed that, for the strongest NBRs, there was a similar proportion of sensorimotor cortex displaying PBR and NBR in the subjects over 80 years old as there was in the subjects under 30. However, in the subjects with the lowest NBR (black dashed lines) we found that much higher proportions of motor cortex showed PBR and also that this dominance of PBR over NBR became even stronger with age, resulting in the PBR/NBR ratio changing from 2 in the Twenties decile to 12 in the Eighties decile. For the subject's in the lower tertile, one-way ANOVA showed a significant effect of decile on PBR (F(1,6)=8.5, *p* = 1 × 10^−7^) and NBR proportion (F(1,6)=8.4, *p* = 2 × 10^−7^) and on the PBR/NBR ratio (F(1,6)=2.3, *p* = 0.02). This suggests that we are not just observing a decrease in NBR with age, but an increasing alteration of the balance between excitation and inhibition in motor cortex across the lifespan. The decline of the magnitude and spatial extent of NBR was associated with increased extent of activation, in some cases with PBR extending into iSM1 in oldest age.

### 3.3 NBR-behavior relationships

Reaction time to the MRI task (mRT) showed no significant relationship with either cSM1 PBR (*R* = 0.007, *p* = 0.84) or iSM1 NBR (*R*=−0.02, *p* = 0.56) Z-statistic. In contrast, both sRT (*R*=−0.09, *p* = 0.04) and cRT (*R*=−0.21, *p* = 9 × 10^−7^) showed significant negative correlations with iSM1 NBR, indicating that RT was shortest in the subjects with largest magnitude NBR. At each of the five phases of the motor learning task, we observed a significant negative correlation between iSM1 NBR and the subject's time taken to hit the target (*R*=−0.18–0.2, all *p*<0.001), indicating that response time was shortest in the subjects with largest magnitude NBR. In the force matching task we did not observe any significant relationships with the NBR magnitude (*R*=−0.05, *p* = 0.08). Although significant NBR-behavior correlations were observed, they were all relatively weak compared to the age-behavior relationships reported above which strongly suggests the NBR-behavior relationships arise indirectly, via a mutual relationship with age. Regression models were run in SPSS to clarify this by showing that adding subject's peak NBR Z-statistic as a regressor could not explain any additional variance in behavioral measures over that already explained by age, and none of the models improved in significance.

### 3.4 Deconvolution to study lifespan changes in HR shape

**Fig. 9:**
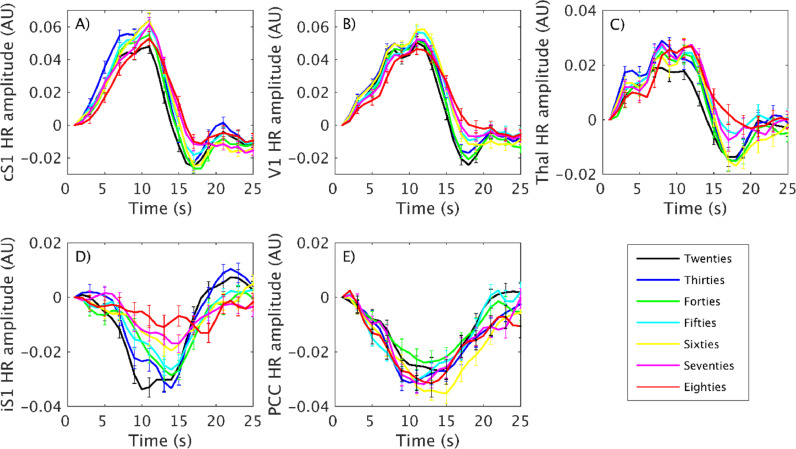
Mean response timecourses of the deconvolved HRs for each decile for cSM1 PBR (A), V1 PBR (B), thalamus PBR (C), iSM1 NBR (D) and DMN PCC NBR (E). Error bars display the standard error in the mean. iSM1 NBR shows progressive decrease in magnitude and increase in time-to-peak between the black (Twenties), blue (Thirties), green (Forties), cyan (Fifties), yellow (Sixties), magenta (Seventies) and red (Eighties) deciles. (For interpretation of the references to color in this figure, the reader is referred to the web version of this article.)

We used a deconvolution analysis to investigate changes in the temporal profile of the BOLD response with aging Fig. 9. plots the mean timecourses of the deconvolved BOLD HRs for each decile group from the three PBR (cSM1, V1 and thalamus; Fig 9A,B,C) and the two NBR (iSM1 and PCC; Fig 9D&E) ROIs. Separately for each ROI and decile we conducted paired t-tests of HR peak magnitudes against zero which showed that all deciles displayed significant cSM1 PBR (all *p*<1 × 10^−20^), V1 PBR (all *p*<1 × 10^−22^), thalamus PBR (all *p*<1 × 10^−17^) and PCC NBR (all *p*<1 × 10^−16^). The iSM1 HR peak magnitude was significantly different from zero (*p*<0.0006) in all deciles except the Eighties decile (*p* = 0.33). The most striking effect of lifespan aging was seen on both the shape and magnitude of the HR from the iSM1 region. In Fig. 9D a steady decrease in NBR magnitude and increase in time-to-peak was seen between each decile, resulting in the NBR becoming progressively shallower and later throughout the lifespan. In contrast both the shape and the magnitude of the NBR from the DMN PCC (Fig. 9E) were highly comparable between deciles and showed no sign of lifespan changes. For iSM1, one way ANOVA, conducted using measures from the single-subject HRs, showed that there was a significant effect of decile on both peak HR magnitude (F(1,6)=3.2, *p* = 0.005) and time-to-peak (F(1,6)=2.2, *p* = 0.03), with iSM1 HR magnitude reducing and peaking later with older age (see Table 3). For the PCC NBR region, one way ANOVA found no effect of decile on either peak HR magnitude (F(1,6)=1.3, *p* = 0.25) or time-to-peak (F(1,6)=0.7, *p* = 0.65) indicating that no changes with age occurred, in agreement with our previous results.

**Table 3:** Measures of lifespan changes in the iSM1 HR peak magnitude and time-to-peak estimated using deconvolution. Data for each decile are shown in individual columns. Data are shown from both the decile group timecourse as well as from single-subject measures, the latter were used to calculate the significance of differences between deciles.

	Twenties	Thirties	Forties	Fifties	Sixties	Seventies	Eighties
**iSM1 peak HR magnitude (from mean decile timecourse)**	−0.034	−0.033	−0.029	−0.027	−0.019	−0.016	−0.013
**iSM1 peak HR magnitude (mean of single subject measures)**	−0.049 ± 0.023	−0.048 ± 0.025	−0.042 ± 0.021	−0.042 ± 0.022	−0.037 ± 0.021	−0.036 ± 0.021	−0.035 ± 0.024
**Significant magnitude difference from Twenties decile? (p value)**	–	No, *p* = 0.88	No, *p* = 0.14	No, *p* = 0.13	Yes, *p* = 0.007	Yes, *p* = 0.006	Yes, *p* = 0.005
**Significantly different magnitude from preceding decile? (p value)**	–	No, *p* = 0.88	No, *p* = 0.20	No, *p* = 0.84	No, *p* = 0.19	No, *p* = 0.98	No, *p* = 0.76
**iSM1 HR time-to-peak (from mean decile timecourse)**	10 s	14 s	14 s	14 s	14 s	15 s	18 s
**iSM1 HR time-to-peak (mean of single subject measures)**	12.2 ± 2.8 s	13.9 ± 3.8 s	14.2 ± 3.8 s	14.2 ± 3.5 s	14.2 ± 4.1 s	14.5 ± 4.6 s	14.5 ± 4.8 s
**Significant temporal difference from Twenties decile? (p value)**	–	Yes, *p* = 0.009	Yes, *p* = 0.005	Yes, *p* = 0.006	Yes, *p* = 0.008	Yes, *p* = 0.003	Yes, *p* = 0.001
**Significant temporal difference from preceding decile? (p value)**	–	Yes, *p* = 0.009	No, *p* = 0.69	No, *p* = 0.96	No, *p* = 0.98	No, *p* = 0.64	No, *p* = 0.98

Post-hoc paired t-tests revealed that lifespan effects manifest differently between the peak magnitude and time-to-peak of the iSM1 NBR (Table 3). The peak NBR magnitude was consistent during the Twenties-Fifties deciles and then significantly declined in the Sixties-Eighties deciles. Whereas the time-to-peak changed much earlier, showing a significant increase between Twenties and Thirties and only small increases during the remainder of the lifespan. This suggests that alterations to the shape of the iSM1 HR occur early in the lifespan whereas decreases in its overall magnitude occur in older age.

In the PBR regions (Fig. 9A–C) the HR peak magnitudes were highly comparable across deciles, with one-way ANOVA showing no significant difference in either cSM1 (F(1,6)=1.6, *p* = 0.18), V1 (F(1,6)=1.01, *p* = 0.42) or thalamus (F(1,6)=1.52, *p* = 0.16) ROIs. ANOVA did find a significant effect of decile on time-to-peak in cSM1 (F(1,6)=3.0, *p* = 0.006) and thalamus (F(1,6)=2.3, *p* = 0.02), but not in V1 (F(1,6)=0.82, *p* = 0.56). The HR time-to-peak increased through the lifespan in both cSM1 (Twenties: 9.4s ± 1.7 s vs Eighties: 10.7 ± 1.9 s) and thalamus (Twenties: 8.2s ± 2.6 s vs Eighties: 10.1 ± 2.8 s).

Fig. 9 and Table 3 provide evidence that the iSM1 NBR was present across the lifespan, it was only in the Eighties decile that peak HR magnitude was not significantly different from zero, but with a reduced and delayed peak magnitude with age. This is in contrast to the group GLM maps (Fig. 1) which showed no significant iSM1 NBR in any of the Fifties-Eighties deciles. These results suggest that the deviation from a canonical HR shape weakens the detection of the iSM1 NBR by the GLM.

### 3.5 GLM analysis using data-driven HR convolution

**Fig. 10:**
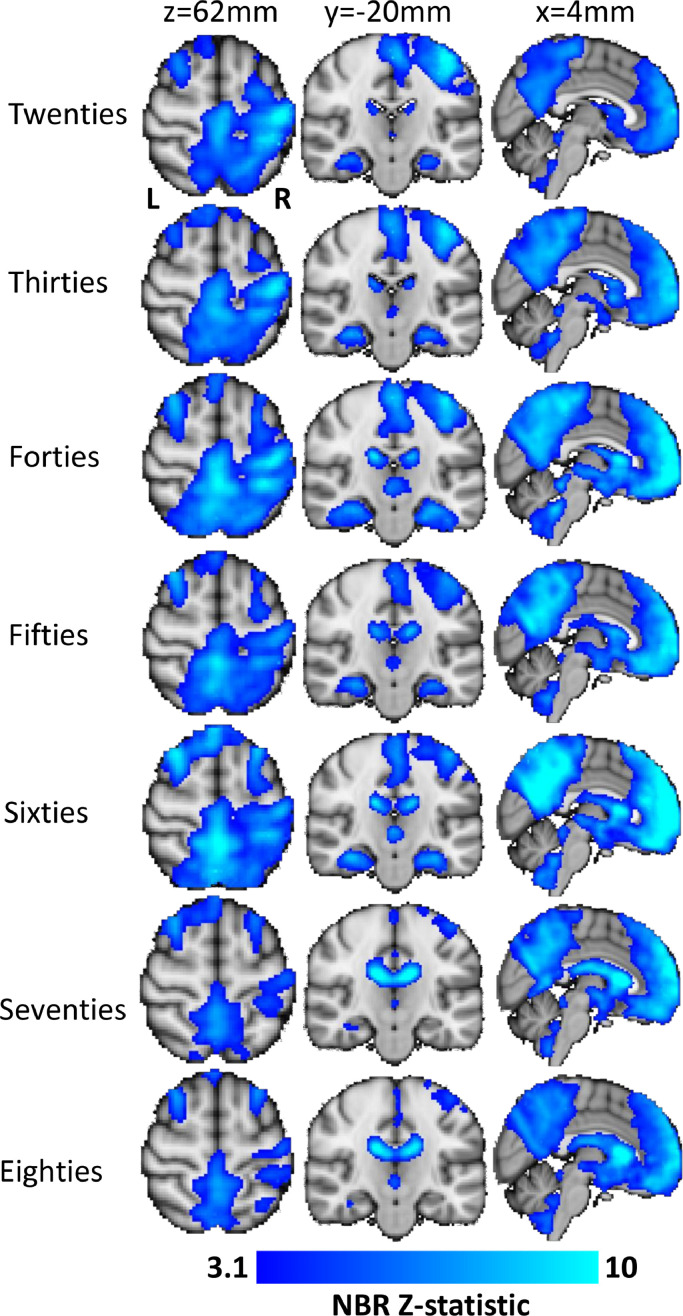
Variation in main-effect NBR across the lifespan calculated using the GLM with the data-driven iSM1 HR obtained from deconvolution. Each row displays the mean NBR (blue) of each the seven decile groups (Twenties-Eighties). Columns 1,2 show axial and coronal slices through the sensorimotor cortex, illustrating how the iSM1 NBR decreases with age. Column 3 shows a sagittal slice through the center of the brain, showing the DMN NBR across the lifespan. (For interpretation of the references to color in this figure, the reader is referred to the web version of this article.)

To further explore how alterations in the shape of the HR affect GLM estimation of iSM1 NBR across the lifespan we performed all GLM analyses again, using the decile mean HRs to perform the convolution of event timings for each subject Fig. 10. shows the average main effect NBR (blue) to the sensory task for each decile group using the data-driven HR. No PBR is shown here as we know from Fig. 9 that the HR used was not appropriate for estimating that response. Significant NBR was observed in iSM1 in all of the decile groups in this analysis, as well as in premotor cortex, supplementary motor cortex and dorsal parietal cortex. The iSM1 NBR was extensive until declining in both spatial extent and magnitude in the Seventies and Eighties. In the Fifties and Sixties the mean first-level Z-statistic was 81% of the Twenties level, which dropped to 74% and 64% respectively in the Seventies and Eighties deciles (Table 2B). Group level peak Z-statistic declined from 10.6 in the Twenties to 5.8 during the Eighties (Table 2B). There was also an indication that the location of the group peak NBR voxel altered slightly between the two analyses, with data-driven peak locations found more dorsal than the canonical responses (Table 2B).

NBR was also observed in the DMN in all decile groups which shows that the HR obtained from one brain region (here iSM1) was able to detect responses in other regions. However, it is not appropriate to infer lifespan effects on DMN NBR from Fig. 10 as the HR used was not optimal for the DMN. We also observed reductions in the BOLD response in the CSF of the ventricles in all deciles which was strongest in the oldest age deciles.

## 4 Discussion

This study provides both the first investigation of how the negative BOLD fMRI response changes across the adult lifespan as well as the most comprehensive assessment to-date of aging effects on multiple NBR characteristics. We used a large (*N* = 581) sample of healthy adults aged 18–88 years performing a motor response task, which extends previous work comparing between young and older adult groups. Our main findings were that the ipsilateral sensorimotor (iSM1) cortex NBR decreased in magnitude and spatial extent throughout the lifespan in a linear manner. The contralateral sensorimotor (cSM1) cortex PBR magnitude and HR shape remained unchanged but the cSM1 PBR did increase in spatial extent with age, reflecting a pattern for greater activation and reduced deactivation both in aging as well as in all subjects that exhibited small NBR. The cSM1 PBR and iSM1 NBR magnitudes were uncoupled in young (<40 years) adults but became negatively correlated in middle and older age, which we suggest also reflects a change in the balance of cortical excitation and inhibition. Furthermore, by using a deconvolution analysis we also found that the shape of the iSM1 HR altered throughout the lifespan, becoming progressively smaller in magnitude and peaking later in time. The most significant decreases in iSM1 NBR magnitude occurred in older age (>60 years) but the first changes in shape and timing occurred much earlier, around 30 years. The age-related deviation of the iSM1 HR from a canonical shape resulted in the conventional GLM analysis significantly underestimating both the magnitude and spatial extent of the iSM1 NBR in subjects as young as their thirties and throughout middle and older age. In contrast we found no significant changes in the magnitude, spatial extent or shape of the DMN NBR. Therefore, the NBR changes in the iSM1 region are the dominant age-related effect observed in this study and our finding of magnitude and time-to-peak effects emerging at different time points in the lifespan suggest there could be more than one contributing factor underlying these alterations. Taken together these results build upon previous evidence of reductions in NBR presentation and disruptions to sensorimotor cortex inhibitory mechanisms with age.

### 4.1 Age-related alterations in the hemispheric balance of excitation and inhibition

A key finding in this study is that we do not solely observe that the iSM1 NBR declines in magnitude and extent with age, but that this decline occurs alongside an increase in the extent of the PBR with the PBR extending into iSM1 in old age in many subjects. Overall, we observed a decrease in the lateralization of the hemispheric BOLD responses and a shift towards more bilateral activation in older age. We further observe that this increased bilaterality of PBR occurs in all subjects with low magnitude NBR, regardless of age (Fig. 8). Interestingly, in the subjects with strongest NBR the balance between PBR and NBR is maintained, whereas in the subjects with the lowest NBR the balance is altered, even in young age, and this disruption worsens considerably across the lifespan (Fig. 8). Therefore, these results suggest an alteration to the balance of functional excitation and inhibition in the motor cortex with age and also that between subject-variability in iSM1 NBR is strongly linked to the extent of activation (PBR). However, it must be noted that precise interpretation of changes in the spatial extent of fMRI responses is made challenging by potential effects of spatial smoothing and the possibility that stronger responses are spread out to a larger extent than small responses by that data preprocessing step.

This finding aligns well with literature on functional neuroimaging in normal aging, which shows a similar age-related increase and bilateralization of activation in older compared to young subjects during motor tasks (Hutchinson et al., 2002; Mattay et al., 2002; Naccarato et al., 2006; Ward and Frackowiak, 2003). The balance of sensorimotor cortex activation becomes less lateralized in aging (Hutchinson et al., 2002; Mattay et al., 2002; Naccarato et al., 2006; Sharma and Baron, 2014) reflecting increasing BOLD signal in both hemispheres. This effect is more pronounced ipsilaterally, manifesting as reduced NBR. Thus, in aging, enhanced M1 recruitment bilaterally is thought to be required to maintain quality of motor performance (Naccarato et al., 2006). Linear increases in activation across the lifespan have been reported in widespread regions outside core processing areas during a semantic judgement task, suggested to represent a compensation mechanism to maintain function (Kennedy et al., 2015). Additionally Brodoehl et al. (2013) detected enhanced activation in the contralateral SM1 and ipsilateral motor cortex in the later stages of somatosensory processing in elderly subjects which they interpret as indicating that, in addition to over-activation to compensate for impaired brain function, there are complex mechanisms of modified inhibition and excitability involved in somatosensory processing in the aging brain (Brodoehl et al., 2013). The hemispheric balance of activity across sensorimotor cortex has been shown to depend on effective transcallosal connections, so we suggest that disruptions to the inhibitory influences that are exerted upon ipsilateral SM1 lead to increased spread of excitation. A high degree of spatial similarity between the cSM1 PBR and iSM1 NBR regions is observed in the current data, including over the hand-knob regions of primary motor cortex as shown in Fig. S2, supporting the possibility of interhemispheric interactions during this task.

### 4.2 Age-related alterations in the BOLD haemodynamic response

To our knowledge this is the first study to investigate age-related changes in the temporal profile of the NBR. Recent studies in visual cortex have reported retinotopic variability of the NBR in young adult visual cortex and that it displayed different temporal dynamics to the PBR (de la Rosa et al., 2021; Puckett et al., 2014). Many studies have investigated age-related changes in the PBR or HRF with largely mixed results. Age-related decreases in PBR magnitude are reported (Ances et al., 2009; Fabiani et al., 2014; Handwerker et al., 2007; Tekes et al., 2005; West et al., 2019) as well as no change in magnitude (Aizenstein et al., 2004; Grinband et al., 2017; Huettel et al., 2001; Rosengarten et al., 2003). Age-related increases in PBR time-to-peak have also been reported (Handwerker et al., 2007; West et al., 2019).

In the current study we found significant age-related differences in both the magnitude and time-to-peak of the HR from the iSM1 region (Fig. 9 and Table 3). HRs from activated sensorimotor, visual cortex and thalamus showed longer response profiles and concordant reductions in the post-stimulus undershoot in older age, as well as increased time-to-peak, but no differences in magnitude.

It is not possible from these data to exactly identify the underlying cause of these HR changes, which could arise from any, and even multiple, stages of the neuro-metabolism-vascular coupling process. Differences in biochemical signaling mechanisms between the different neural events underlying PBR and NBR could stimulate different vasodilation properties and haemodynamic responses. However, given that we observe age-related changes in HR amplitude and shape that are specific to NBR in iSM1 we suggest that this originates from age-related changes in the iSM1 neural input rather than local alterations to the HRF. Whilst advancing age is linked to both gray matter atrophy (Giorgio et al., 2010; Good et al., 2001) and detriments to cerebral vascular responses (Ances et al., 2009; Liu et al., 2013; Lu et al., 2011) these are reported as generally global effects and it seems unlikely that sufficiently differential effects could occur between left and right motor hemispheres that could explain our results in terms of HRF changes alone. This, together with our observation of no age-related effects upon the DMN NBR, suggest that the iSM1 NBR is generated by different mechanisms to the iSM1 NBR and that it isn't a cortex-wide process that is affected by age, but one that is specific to the sensorimotor system, the most likely being a change in inter-hemispheric signaling via the corpus callosum as is further explored below.

Overall, we found that GLM re-analysis by convolving the stimulus timings with a data-driven, decile-specific HR as a substitute for the canonical HRF showed much greater continuity of the magnitude (Z-statistic) and spatial extent of the iSM1 NBR throughout the lifespan (Fig. 10) than was observed in the canonical HRF GLM (Fig. 1). We used this HR as a custom model to improve sensitivity for detection of a specific response. It is important to be clear that this is not the same as using a custom HRF and it would not be appropriate to use this HR as a convolution kernel for anything other than a delta function in motor cortex with a button press task. There is also an element of circularity in this analysis. Ideally a secondary dataset would be used to fully determine the HR, which would then be applied in analysing the study data, but that was not available here. However, our findings do have implications for studies of age-related changes in fMRI signal, as we find that changes in the shape of the NBR with age led to substantial underestimation of the magnitude and spatial extent of the iSM1 NBR for age groups over 40 years old (Fig. 1 cf Fig. 10.). Reanalysis using convolution with a data-driven HR revealed iSM1 NBR was present throughout the lifespan, even as late as the Eighties decile (Fig. 10), which was not detected by a GLM using the canonical double-gamma HRF. This suggests that deviation of NBRs from the canonical HRF shape can substantially confound their accurate measurement in aging and that data-driven methods are required for accurate assessment of lifespan effects on NBR. This observation is borne out when comparing our finding that a NBR is present in most deciles with those of previous, small sample, studies that used a conventional canonical HRF and reported disappearance of ipsilateral sensorimotor NBR >70 years of age (Groschel et al., 2013; McGregor et al., 2009; Riecker et al., 2006). Future studies should consider employing means to estimate HRs or HRFs, as study appropriate, to ensure accurate measurement of BOLD, and especially NBR, changes with age. Given that we observe alterations in NBR shape even within young adults our findings have implications for all adult studies of NBR, not just aging studies.

### 4.3 Changes in inhibition throughout the lifespan

Our findings are in agreement with previous fMRI studies which showed a decline in sensorimotor NBR magnitude in older age (Groschel et al., 2013; McGregor et al., 2009, 2015; Naccarato et al., 2006; Riecker et al., 2006; Ward et al., 2008). Our work both replicates and considerably extends the literature as most previous studies involved comparisons between relatively small (*N* ≈ 20) groups of young and old adults, whereas the current work utilises a dataset of 581 right-handed subjects evenly distributed (18–88 years) in age which enabled us to study changes in NBR throughout the adult lifespan and identify when alterations in shape, timing and magnitude arise. We observed that aging was associated with a linear decrease in the visual cortex PBR and an increase in the time-to-peak of cSM1 and thalamus PBR. We did not observe any significant age-related differences in the magnitude, spatial extent or shape of the DMN NBR. This finding is in contrast to previous work which showed that the magnitude and spatial extent of DMN NBR significantly decreased between groups of young and older adults performing semantic classification, episodic and working memory tasks (Grady et al., 2006; Lustig et al., 2003; Sambataro et al., 2010). This has been suggested to reflect a progressive age-related decline in the suspension of non-task-related activity and the ability to engage task processing areas (Grady et al., 2006). However, we suggest our findings could arise due to the different requirements between the simple sensory response task used here, which was not behaviorally challenging (hence >98% accuracy) and the cognitively demanding memory tasks that are employed in most previous studies. DMN deactivation is well known to be manipulated by the paradigm. DMN NBR magnitude has been shown to increase with the level of task demand (McKiernan et al., 2003; Singh and Fawcett, 2008) and the DMN is a not a homogenous network but shows complexity in the spatial pattern of its responses to different tasks and in its functional connections (Harrison et al., 2008; Leech et al., 2012, 2011). A recent study of 117 adults aged between 25 and 83 years found a linear decline in DMN NBR with age during a difficult task-switching condition, and that this relationship was mediated by age-related decreases in white matter microstructure. However, they observed no age-related decrease in NBR during a simpler condition of the task which is attributed to much lower cognitive control being required (Brown et al., 2015). A further recent study featuring slow flexions of the right wrist also report no alteration in DMN NBR between groups of young and older adults (Morita and Naito, 2021). Our results conform with this previous work and suggest that for tasks featuring low levels of cognitive control the deleterious effect of aging on the DMN response appears to be minimal. However, whilst we do not observe any clear effect of age upon the DMN NBR we did observe a clear relationship between iSM1 and DMN NBRs which may suggest the existence of a general relationship between total excitation and inhibition in the brain, where the subjects with weaker iSM1 NBR also display weaker DMN NBR regardless of the task difficulty.

Under the assumption that NBR reflects, at least partly, a functional measure of cortical inhibition (Kastrup et al., 2008; Mullinger et al., 2014; Schafer et al., 2012; Sten et al., 2017), any attempts to understand the neurophysiological basis of age-related changes in NBR should consider the considerable magnetic resonance spectroscopy (MRS) and transcranial magnetic stimulation (TMS) literature that has studied alterations in the primary inhibitory neurotransmitter, gamma-aminobutyric acid (GABA), and mechanisms of cortical inhibition with aging. MRS studies generally find a linear reduction in cortical GABA levels with age (Gao et al., 2013; Porges et al., 2021, 2017) and although data from sensorimotor cortex can appear less conclusive, reporting both decreases (Cuypers et al., 2020; Grachev et al., 2001; Maes et al., 2021) and no change in older age (Hermans et al., 2018; Mooney et al., 2017) there is an emerging consensus that older adults exhibit a reduced capacity to modulate GABA-ergic inhibition which is associated with degraded motor performance (Levin et al., 2014; Pauwels et al., 2019).

Aging has been reported to reduce the size of the increase in interhemispheric inhibition (IHI) that occurs during performance of motor tasks (Talelli et al., 2008b), suggesting that aging attenuates the amount of inhibition that is targeted on the ipsilateral cortex during right-hand activity. Additionally, between-subject measures of IHI were found to explain variability in ipsilateral motor BOLD responses beyond that explained by age (Talelli et al., 2008a) such that those subjects with decreased ability to modulate IHI processes displayed weaker iSM1 NBR (and increased bilaterality of PBR). Boudrias et al. (2012) combined TMS measures of IHI with dynamic causal modeling of fMRI data and found that interhemispheric signaling between cM1 and iM1 showed reduced inhibition or greater facilitation in older adults which enabled maintained quality of task performance compared to young adults. This suggests that aging is associated with weakening of interhemispheric inhibition in motor cortex, arising from decreased excitability of interhemispheric connections or degradation of transcallosal fibres, as evidenced by the disappearance of ipsilateral NBR in patients with collosal agenesis (Genc et al., 2015). Therefore, despite a general lack of previous data from across the lifespan, there is evidence for age-related decreases in interhemispheric inhibitory processes, and reduced transcollosal excitation, which could underlie the changes in NBR that we observe here.

We also observed an age-related reduction in the magnitude of the post-stimulus undershoot in cSM1, V1 and thalamic PBR regions. The undershoot was pronounced in the youngest deciles but largely absent in the oldest deciles. A consequence of this was that the duration of the primary PBR became slightly longer in older age. This change is interesting since the post-stimulus response is also thought to be primarily driven by inhibitory neuronal activity and to involve a different balance of inhibition and excitation to that of the primary PBR (Mullinger et al., 2017, 2013). Therefore, the observed reduction of the post-stimulus response with age may relate to the change in the NBR, with both potentially reflecting age-related altered inhibitory neuronal mechanisms. Post-stimulus responses have been hypothesised to be generated by the re-integration of parts of resting state brain networks which had diverged their activity to perform the task (Mullinger et al., 2017, 2013; Tewarie et al., 2019). In this case the white matter fiber tracts and transcollosal connections would be as vital to the generation of the post-stimulus response as they are for the iSM1 NBR. This work therefore also provides new evidence which supports the nascent hypotheses about the role and importance of the post-stimulus response and requires further investigation.

### 4.4 Functional relevance of decreased sensorimotor NBR in aging

We found no relationship between sensorimotor BOLD responses and subject's reaction time to the MRI task, which may appear surprising. We also find no effect of age upon subject's reaction time, which contrasts with reports of age-related decline of perceptual speed (Fozard et al., 1994; Salthouse, 1996). However, we suggest this may be explained by the MRI task not being delivered as a test of reaction time, but more as a means of acknowledging that the audiovisua stimulus was perceived, hence the significant relationship we see between NBR and both sRT and cRT (measured in separate sessions) is not repeated in the MRI data. In addition, as neither task accuracy or reaction time showed any age dependent effects, we suggest that the alterations in iSM1 NBR that we observe occur independently from differences in task behavior and that such differences would become more pronounced if assessed under conditions that demonstrated age-related alterations in behavior. Whilst we observed that NBR was correlated with various behavioral measures of sensorimotor function, NBR explained much less behavioral variance than subject's age and could not contribute additional power to regression models containing age. Due to the inter-correlation between these variables it is difficult to determine NBRs potential links to behavior and its role, if any, in sensorimotor function. However, as age-related decreases in sensorimotor inhibition are generally associated with degradation of motor performance (Cuypers et al., 2020; Levin et al., 2014; Maes et al., 2021; Mattay et al., 2002; Pauwels et al., 2019) further work should expand on suggested links between behavior NBR and interhemispheric GABA-ergic inhibition (Talelli et al., 2008b) to elucidate this relationship.

### 4.5 NBRs from ventricular CSF

In both GLM analyses we observed NBRs in the CSF of the ventricles and a tendency for the magnitude and spatial extent of this NBR to increase with age (see Figs. 1 and 10). NBR in the ventricles are not widely reported but have been investigated by a few studies (Bianciardi et al., 2011; Bright et al., 2014; Thomas et al., 2013; van der Zwaag et al., 2009). It is not thought that such NBR, arising as they do in areas devoid of neural tissue, originate from comparable mechanisms to the DMN and sensory cortex NBR that are the main focus of this paper. Whilst the BOLD signal is determined by the ratio of oxygenated to deoxygenated blood, which is what gives rise to its ability to map brain function, it is also strongly influenced by changes in cerebral blood volume (CBV) that can occur without changes in blood oxygenation and can be entirely unrelated to neural activity (Bianciardi et al., 2011; Buxton et al., 2004). As such these ventricle responses represent spurious anticorrelated activity due to increases in CBV in large cerebral draining veins that act to decrease local T_2_* signal and appear as a NBR in the ventricles (Bianciardi et al., 2011). These NBRs are typically observed around the edges of the ventricles, where such veins lie. These results provide a reminder that NBR can arise from purely vascular mechanisms (Harel et al., 2002) as has also been observed due to venous CBV effects amidst cortical gray matter (Olman et al., 2007; Puckett et al., 2014). We observed NBR throughout the entire ventricles in the oldest subjects, which may indicate that such CBV effects get stronger with age, perhaps due to the greater extent of the PBR. These effects may be compounded by spatial smoothing effects and slight registration inaccuracies due to shrinkage of gray matter and ventricular enlargement with age (Fjell and Walhovd, 2010).

## 5 Conclusion

This study has found that ipsilateral sensorimotor NBR and DMN NBR, to a simple sensory task, are differentially affected by aging across the lifespan. iSM1 NBR exhibits changes in shape and increases in peak latency from young age throughout adult life, which are accompanied by decreases in NBR magnitude in older age. The alterations to the temporal profile of the NBR result in considerable underestimation of the iSM1 NBR in older age by a GLM using a canonical HRF. The sensorimotor PBR and NBR magnitudes are uncoupled in young adults but become correlated in middle and older age, coincident with reduced lateralization of the two responses whereby the spatial extent of NBR decreases as activation becomes more bilateral in older age. In contrast DMN NBR was not observed to alter in magnitude, spatial extent or shape at any point in the adult lifespan. Taken together our results suggest that sensorimotor inhibitory control changes as a function of age, as age-related changes in iSM1 neural input are consistent with: (1) the declining amplitude of the NBR but unaffected PBR; (2) the changes in spatial distribution of the NBR but not PBR; (3) the specificity of the change in iSM1 NBR during motor processing with no change to NBR in the DMN, (4) the age-related changes in RTs. Therefore, our fMRI measures may reflect previously reported decreases in transcollosal inhibition that occur with age alongside alterations to the excitatory-inhibitory balance in the sensorimotor network.

## Data and code availability statement

All data used in the preparation of this work were obtained from the Cambridge center Aging and Neuroscience data set (Cam-Can repository, and are freely available at http://www.mrc-cbu.cam.ac.uk/datasets/camcan/). Code used to process data and prepare figures is available on request from the corresponding author (s.d.mayhew@bham.ac.uk).

## CRediT authorship contribution statement

**Stephen D. Mayhew:** Formal analysis, Writing – original draft. **Sebastian C. Coleman:** Formal analysis. **Karen J. Mullinger:** Writing – original draft. **Cam Can:** Visualization, Data curation.

## Appendix Supplementary materials

Fig. S1. Histogram of the age of the 581 right-handed subjects used in this study, showing the even distribution of age across all the deciles analysed. Image, image 1

Fig. S2. Group-level example of the spatial homology between hemispheres of the cSM1 PBR and the iSM1 NBR using the data from the Twenties decile. The mean NBR (blue) is shown with the mean PBR (red/yellow) reflected in the x-axis so that the PBR displays in the ipsilateral (right) hemisphere. The conjunction of PBR and NBR is plotted in green. Image, image 2
