# Supplementary figures and images for: Across the adult lifespan the ipsilateral sensorimotor cortex negative BOLD response exhibits decreases in magnitude and spatial extent suggesting declining inhibitory control
Source: Neuroimage. 2022 Jun;253:119081. doi: 10.1016/j.neuroimage.2022.119081 (PMC9130740; doi:10.1016/j.neuroimage.2022.119081)

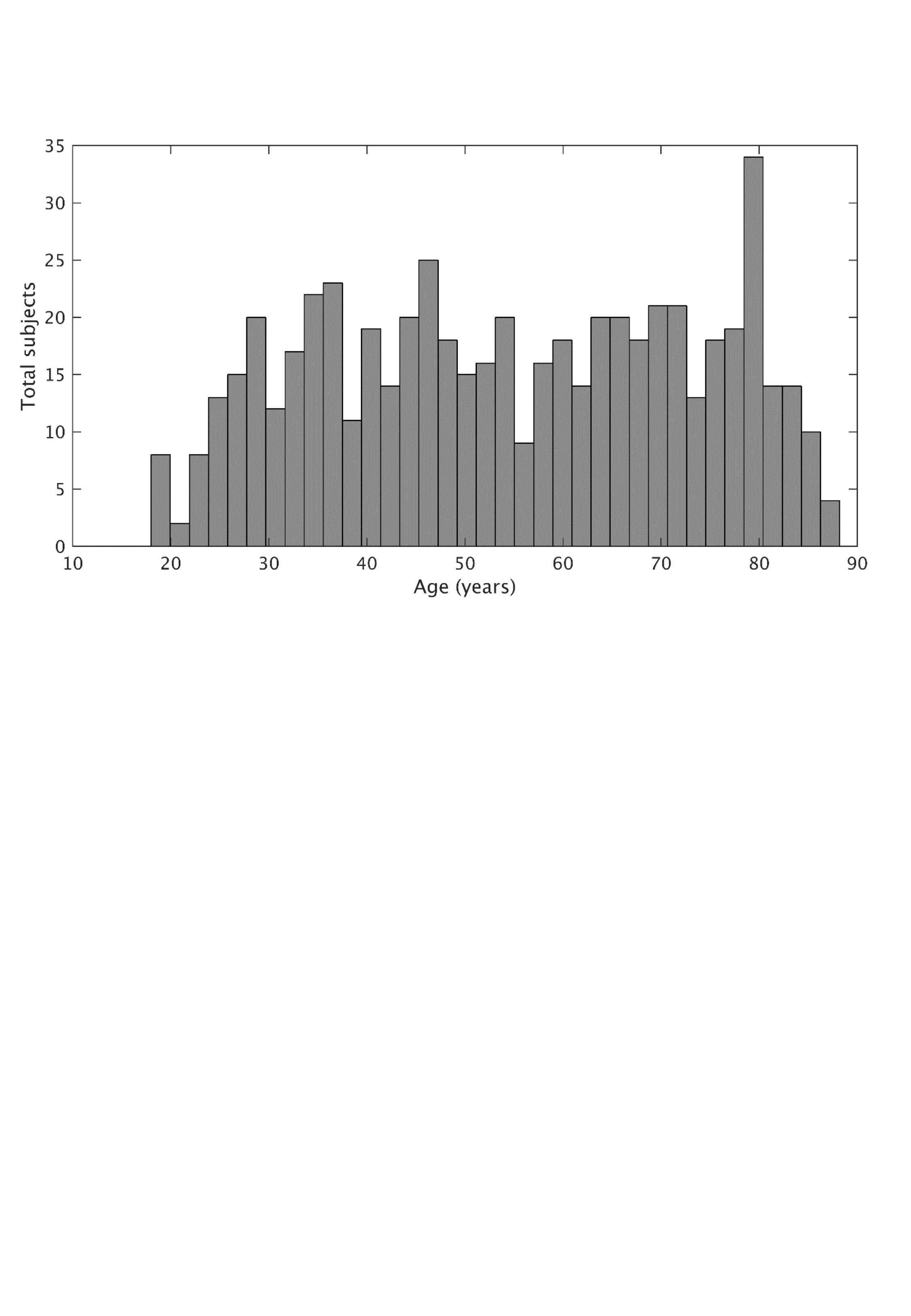

Supplement: Supplementary file 1 — Fig. S1. Histogram of the age of the 581 right-handed subjects used in this study, showing the even distribution of age across all the deciles analysed. [file mmc1.zip › mmc1.tif]

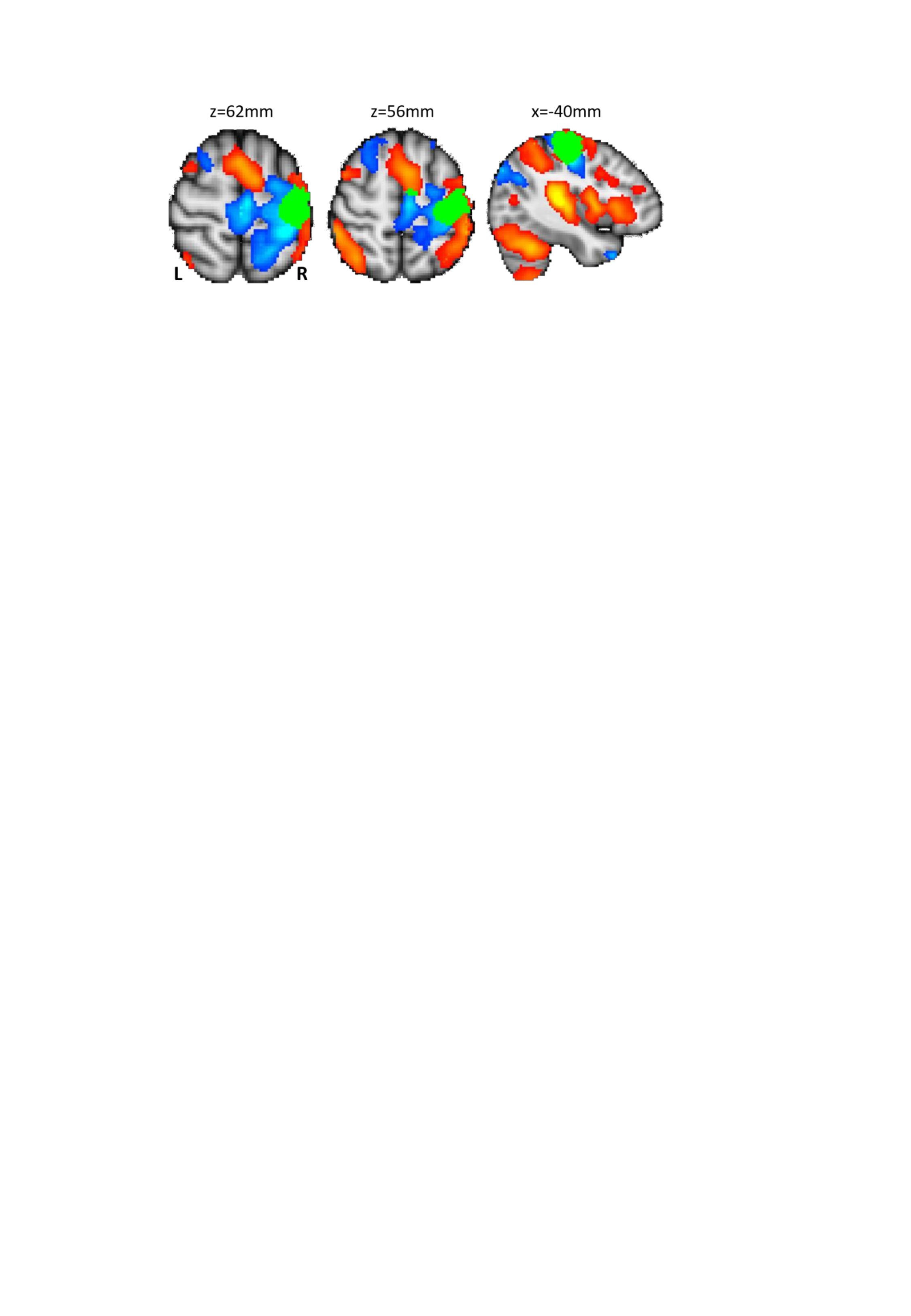

Supplement: Supplementary file 2 — Fig. S2. Group-level example of the spatial homology between hemispheres of the cSM1 PBR and the iSM1 NBR using the data from the Twenties decile. The mean NBR (blue) is shown with the mean PBR (red/yellow) reflected in the x-axis so that the PBR displays in the ipsilateral (right) hemisphere. The conjunction of PBR and NBR is plotted in green. [file mmc2.zip › mmc2.tif]
